# Pregabalin in the treatment of inferior alveolar nerve paraesthesia following overfilling of endodontic sealer

**DOI:** 10.4317/jced.51420

**Published:** 2014-04-01

**Authors:** Oscar Alonso-Ezpeleta, Pablo J. Martín, José López-López, Lizett Castellanos-Cosano, Jenifer Martín-González, Juan J. Segura-Egea

**Affiliations:** 1 Associate Professor. Department of Endodontics, School of Dentistry, University of Zaragoza, 22006-Huesca, Spain; 2 Doctoral fellow. Department of Stomatology, School of Dentistry, University of Sevilla, C/ Avicena s/n, 41009-Sevilla, Spain; 3 Professor. Department of Odontostomatology, University of Barcelona, Barcelona, Spain; 4 Full Professor. Department of Endodontics, School of Dentistry, University of Sevilla, C/ Avicena s/n, 41009-Sevilla, Spain

## Abstract

A case of orofacial pain and inferior alveolar nerve (IAN) paraesthesia after extrusion of endodontic sealer within the mandibular canal treated with prednisone and pregabalin is described. A 36-year-old woman underwent root canal treatment of the mandibular second right premolar tooth. Post-operative panoramic radiograph revealed the presence of radiopaque canal sealer in the mandibular canal. Damage to IAN consecutive to extrusion of endodontic sealer was diagnosed. Non-surgical management was decided, including: 1 mg/kg/day prednisone 2 times/day, once-daily regimen, and 150 mg/day pregabalin, two doses per day, monitoring the progress with periodic follow-up visits. Six weeks after the incident the signs and symptoms were gone. The complete resolution of paraesthesia and the control of pain achieved suggest that a non-surgical approach, combining prednisone and the GABA analogue pregabalin, is a good option in the management of the IAN damage subsequent to endodontic sealer extrusion.

** Key words:**Endodontics, inferior alveolar nerve, neuropathic pain, orofacial pain, paraesthesia, pregabalin.

## Introduction

Injuries of the inferior alveolar nerve (IAN), represent a rare but serious complication of dental treatment (1). Overinstrumentation during root canal treatment with manual or rotary instruments can perforate the mandibular canal. This allows the extrusion of endodontic sealers, dressing agents, and irrigation solutions out of the tooth and into the canal that may lead to IAN injury (2). Although small material extrusions are generally well tolerated by the periradicular tissues (3), clinical symptoms are disabling sensory disturbances such as pain, dysesthesia, paresthesia, hypoesthesia, and anesthesia, which have been reported after the extrusion of endodontic materials into the mandibular canal. Almost all of the endodontic materials are neurotoxic at some level and are able to initiate a inflammatory process which causes damage to cells and might culminate in necrosis of the tissue (3,4,5,6,7,8,9).

Paresthesia might be caused by several factors, both local and systemic. Local factors are, for example, the use of local anesthetics, trauma, local infections, neoplasia, tooth extraction, and endodontic related complications. Systemic factors are, for example, microbial infections, multiple sclerosis, lymphoma, or diabetes mellitus (4,10).

The normal therapeutic sequence for injuries to IAN is the control of pain and inflammation and, whenever possible, the surgical elimination of the cause (11). However, total resolution of pain and reduction or disappearance of paraesthesia after a non-surgical management have been reported (3,7,8,9,12).

Pregabalin is a GABA analogue with similar structure and actions to gabapentin. It has antiepileptic, analgesic and anxiolytic activity. Its ability to reduce neurotransmitter release from activated neurons in pain pathways and fear circuits may contribute to its role as an adjuvant in pain management and as anxiolytics. Pregabalin has proven effect in chronic and neuropathic pain (13-15).

The aim of this paper is to describe a case of endodontic sealer penetration within the mandibular canal after root canal treatment of a mandibular right second premolar, with resolution of pain and paraesthesia after a non-surgical approach, including treatment with prednisone and pregabalin.

## Case Report

**Figure 1:**
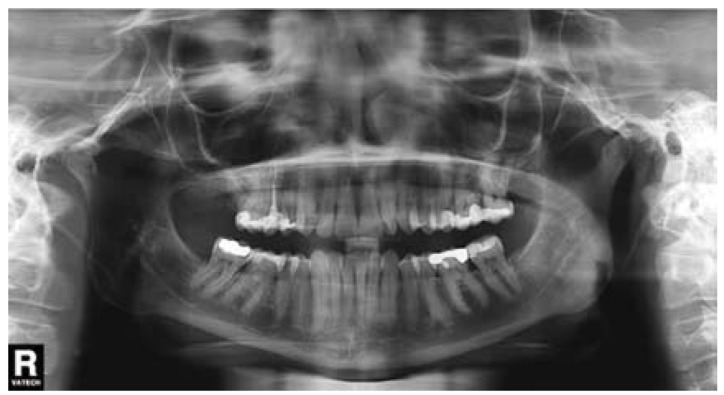
Panoramic radiograph revealed widening of periodontal ligament space in the root apex of the right mandibular second premolar.

**Figure 2:**
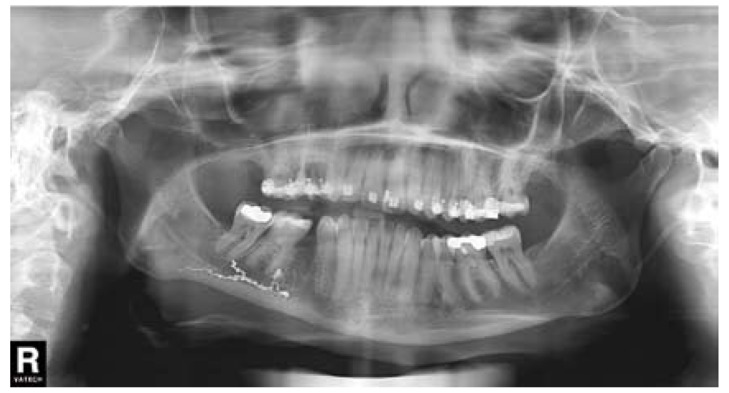
Panoramic radiograph revealing the presence of radiopaque material (endodontic sealer) within the mandibular canal. Tooth 45 has been extracted.

A 36-year-old woman requested dental attention to her dentist. The main complaints were pain and swelling in the right mandibular second premolar (#45). Panoramic radiograph revealed widening of periodontal ligament space in the root apex of the affected tooth (Fig. 1). Acute apical periodontitis subsequent to caries was diagnosed and root canal treatment was carried out. One day after endodontic treatment, the patient had severe pain in the treated tooth and numbness on the right side of the lower lip that extended from the mandibular midline to the second premolar. Her dentist prescribed ibuprofen during one week, but after that time, pain and paresthesia persisted, without any kind of improvement. The extraction of the right mandibular second premolar tooth was decided. Two weeks after extraction, pain had not completely disappeared, and paresthesia remained. Panoramic radiograph showed radiopaque material within the mandibular canal (Fig. 2). The dentist diagnosed neuropathic pain and inferior alveolar nerve paresthesia after extrusion of endodontic sealer and then, decided to refer the patient to the University Dental Clinic.

The referring dentist communicated that the root canal treatment was done with adequate anaesthesia and isolation with rubber dam. The endodontic access cavity was prepared with round diamond burs, Komet 014 (Gebr. Brasseler GmbH, Lemgo, Germany). One canal orifice was found. Apical patency was carried out, and working length was determined electronically using Dentaport ZX (J. Morita Mfg. Corp. Higashihama Minamicho, Fushimi-ku, Kyoto, Japan). The root canal was irrigated with 0,2 % chlorhexidine (CHX) and instrumented with Reciproc R25 (VDW GmbH, Munich, Germany). After cleaning and shaping, the canal was dried and filled with AH Plus Jet (Dentsply DeTrey GmbH, Konstanz, Germany) and gutta-percha, using the lateral compaction technique. A small quantity of sealer was introduced into the root canal using an intra-oral adjustable tip attached with this sealer. Then the main gutta-percha cone was placed, coated with a minimal quantity of sealer. Cold compaction was had performed with a digital spreader and auxiliary gutta-percha cone which was covered with a small quantity of sealer.

**Figure 3:**
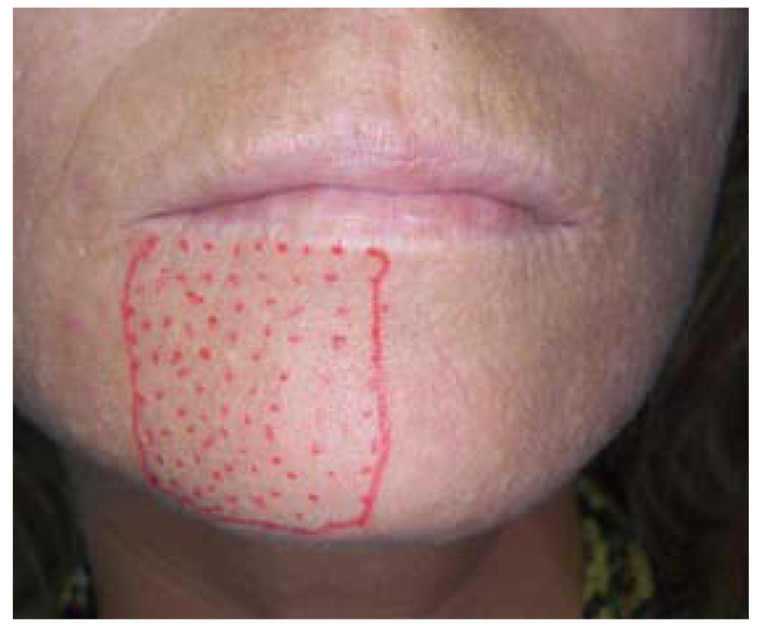
Three weeks after the accident during the root canal treatment, a delimitation of the mental nerve anaesthesia is performed.

The anaesthetized zone in the region innervated by the right inferior alveolar nerve was delimited by tactile exploration with an explorer (Fig. 3). Neither buccal gingival tissues nor the lower lip between the midline and the second premolar showed sensitivity to thermal or mechanical stimuli.

After discussing treatment options with the patient, who refused surgical debridement of the mandibular canal and decompression of the inferior alveolar nerve, a non-surgical approach was therefore decided. Treatment started with anti-inflammatory regimen including 1 mg/kg/day of prednisone (Dacortin®, 30 mg; Merck SL, Madrid, Spain) in two doses, in a gradually reducing regimen on a daily basis, and 150 mg per day of pregabalin (Lyrica®, 75 mg; Pfizer SL, Barcelona, Spain), two doses by day, monitoring the progress with periodic follow-up visits.

**Figure 4:**
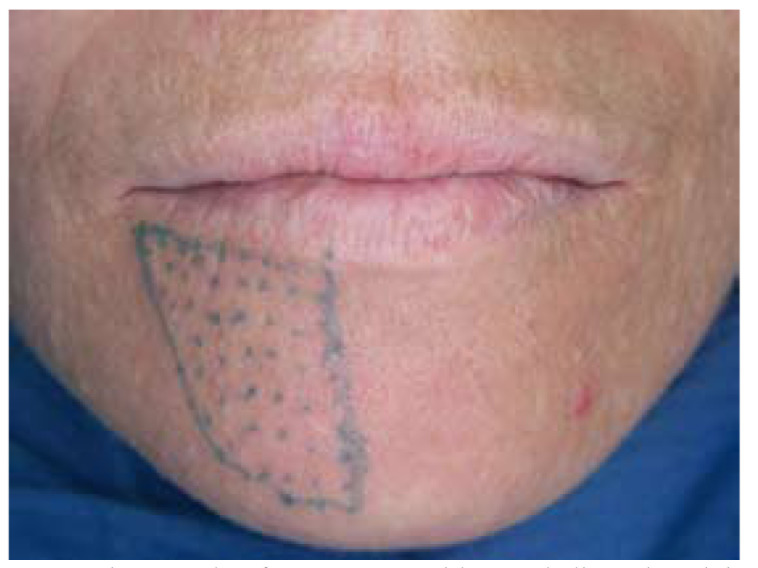
Three weeks after treatment with pregabalin and prednisone the area of mental nerve anaesthesia has decreased substantially.

**Figure 5:**
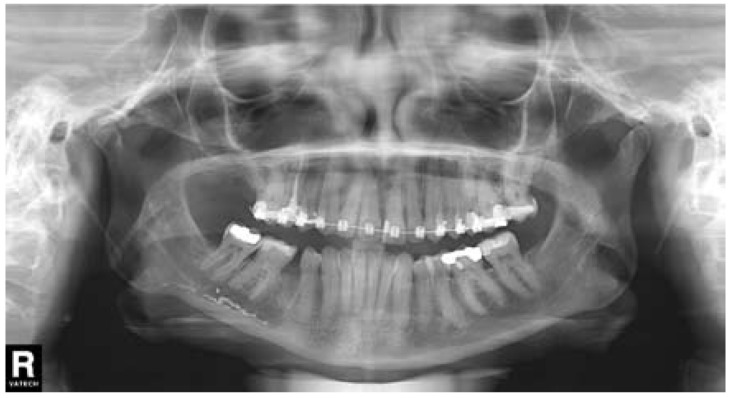
Panoramic radiograph after three weeks of treatment with pregabalin and prednisone.

**Figure 6:**
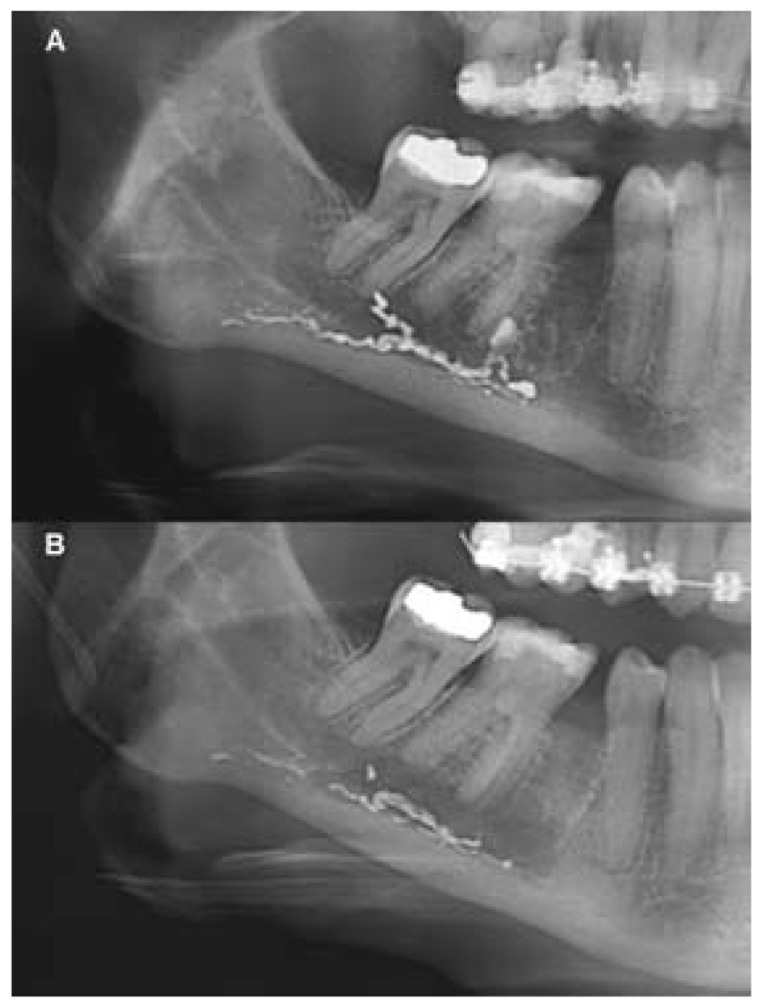
A) Initial panoramic radiograph detail. B) Panoramic radiograph detail after three weeks of treatment. A significant reduction in the amount of endodontic sealer within the mandibular canal is observed.

The patient noticed a significant improvement in the first days after the medical treatment. One week later, the patient had no pain, and paraesthesia in the region of the right lower lip had decreased more than half of its surface. The prednisone was stopped but the pregabalin regimen was maintained. Three weeks later, the paraesthesia was reduced substantially compared with the initial situation (Fig. 4) and pregabalin treatment was finished. Panoramic radiograph was taken, revealing a significant reduction in the amount of endodontic sealer within the mandibular canal (Fig. 5,6). The patient reported a gradual reduction of paraesthesia and after six weeks signs and symptoms had disappeared.

## Discussion

A case of neuropathic pain and inferior alveolar nerve paresthesia after extrusion of endodontic sealer within the mandibular canal has been described. Most of published cases of paresthesia related to endodontics might be caused by overinstrumentation and/or overfill of endodontic materials into the mandibular canal (1,10). IAN injuries have been reported in relation with endodontic treatment in second mandibular molars, because the distance between the apexes and the mandibular canal is often less than 1 mm. However, first molars and premolars must be also taken into consideration (11,16,17).

Overinstrumentation and/or overfilling of mandibular molar and premolar are a potential iatrogenic cause of IAN injury (18). Gutta-percha is traditionally considered an inert root-filling material, and the paresthesia cases involving gutta-percha usually result from overfill of thermoplastic gutta-percha. If the sealer is extruded in the mandibular canal space, it can cause problems that vary from mild inflammatory reactions to severe neurotoxic damage (11,19,20).

Severe endodontic pain after endodontic sealer extrusion requires early diagnosis and prompt management to reduce the risk of permanent nerve damage. Cone-beam computed tomography (CBCT) is often useful in monitoring the area of tissue damage in relation to hard tissues (eg. the location of extruded material in relation to mandibular canal or mental foramen) (21,22).

The literature describes four possibilities of endodontic sealer spreading to the periapical region: toward the mandibular canal, drainage through lymphatic vessels, systemic diffusion through a periapical vein and progression toward soft tissues between bone and mucosal membrane (3,23). The case reported here corresponds with the first of these routes.

The first symptom of overextension into the mandibular canal is sudden pain during filling of the root canal, which persists after the disappearance of the local anaesthetic (24). The literature provides ample evidence that this might induce mild or serious transient inflammatory responses (25,26). Due to this, pain can be accompanied by local inflammatory signs with painful tooth to percussion, painful upon palpation of the buccal alveolar process or a combination of signs of mechanical trauma and inferior dental nerve inflammation with pain or numbness of the lower lip or otalgia (27). Some patients experience persistent anaesthesia (1,10,28,29). In the present case, symptoms occurred after one day, and pain was associated with paraesthesia.

Damage by accidental extrusion of sealer into the mandibular canal may occur due to mechanical, thermal, or chemical processes (4,6,9,30). In the case described here, AH Plus Jet extruded into the mandibular canal. This sealer is commercialized as a syringe of automix, with a final cannula that introduces it into the canal. This sealer is commercially as a syringe of automix, with a final cannula that introduces it into the canal. It is an open-end cannula, therefore it could be applied the same as the irrigation needles. Lots of bibliographic sources conclude that these have higher probability of apical extrusion (31,32,33). Because of the use of this mechanism in the inclusion of cement on the root canal, it was able to facilitate its extrusion by the apex.

AH Plus Jet is a commonly used epoxy resin-based root canal sealer with the monomer 2,2-bis(4-(2-hydroxy-3-methacrylyloxypropoxy) phenyl)-propane (BisGMA), prepared from bisphenol A and glycidyl methacrylate, as its major ingredient (34). Previous reports have shown that AH Plus can cause cytotoxic effects (35) and neurotoxicity when extruded into the mandibular canal (8). Moreover, it has been shown that its component bisphenol A can also cause cytotoxicity (36). The cytotoxic effect of AH Plus has been shown to be dependent on the setting time, showing a significant reduction after 7 days (37). In a recent study of the cytotoxicity of AH Plus, there was no evidence for DNA double-strand breaks caused; so far, there are no reports in the literature on the occurrence of periapical malignant lesions that might be caused by the extrusion of root canal sealer (38).

In this case, polymerization of the sealer may explain, at least in part, the rapid improvement observed in the first days after the incident. Gutta-percha and sealers may cause both an inflammatory reaction and pain when extruded beyond the root canal system. The nature and degree of tissue reaction is related to the type and amount of sealer, the location of the extrusion, and the condition of periodontal tissues (30). Even root canal sealants that are believed to be more benign, such as zinc oxide, eugenol, and calcium hydroxıde (owing to high pH), have been shown to be neurotoxic in vitro and are more certainly neurotoxic in vivo (40). In some experimental studies it was shown that paraformaldehyde is a polymeric hydrate of formaldehyde, which when in contact with water releases formaldehyde gas, and may cause permanent damage to the nerve (41).

Treatment of this endodontic complication remains controversial, varying from a wait-and-see approach, including anti-inflammatory drugs and periodic follow-up (3,7,8,9,12), to early, if not immediate, surgical debridement of the inferior alveolar nerve involving bone removal of the vestibular cortical plate (2,6,18) or sagittal mandibulotomy (19). The patient refused surgical debridement of the inferior alveolar canal and decompression of the inferior alveolar nerve, and decided to make the extraction of second lower right premolar. So, a non-surgical management was agreed including anti-inflammatory treatment with prednisone and analgesic treatment with pregabalin.

Most of the pain treatments used for neuropathic pain have not been approved by FDA, including all the tricyclic antidepressants and most of the anticonvulsants. Two medications are approved for peripheral neuropathy by the FDA; duloxetine and pregabalin (42). In July 2004 pregabalin was granted approval in all European member states for the treatment of peripheral neuropathic pain. Pregabalin is an analogue of the inhibitory neurotransmitter gamma-aminobutyric acid (GABA). Although its main indication is chronic pain and trigeminal neuropathic pain (14,43), it has been frequently used in neuropathic pain (44). Pregabalin has shown analgesic activity in preclinical and clinical models, observing a significant improvement as early as week 1 and is maintained throughout the period of treatment (45,46) and appears to have significant analgesic properties following third molar extraction (47). Peak plasma levels occur approximately 1 hour after oral doses and oral bioavailability is approximately 90%. Pregabalin is not protein-bound and exhibits a plasma half-life of 6 hours, which is not dose-dependent. These antiepileptic drugs have a favorable safety profile with minimal concerns regarding drug interactions and showing no interference with hepatic enzymes (48). Hepatic metabolism is negligible, and most of the oral dose (95%) is eliminated by renal excretion. Pregabalin is a safe and well-tolerated new treatment for neuropathic pain and the most common side effects included dizziness, and somnolence, peripheral edema, weight gain, and asthenia, but this finding was not reported in this case (13,49). Taking into account that endodontic sealer extrusion into the mandibular canal damages the inferior alveolar nerve triggering neuropatic pain, the use of pregabalin in the case reported here was justified.

The complete resolution of the paraesthesia and the control of pain achieved in the present case, suggests that a non-surgical approach combining prednisone and pregabalin is a good option in the management of inferior alveolar nerve damage subsequent to endodontic sealer extrusion. If medical treatments have failed, invasive therapies such as intrathecal drug administration and neurosurgical techniques may be considered.
